# Supplementary figures and images for: Transcriptome response to elevated atmospheric CO2 concentration in the Formosan subterranean termite, Coptotermes formosanus Shiraki (Isoptera: Rhinotermitidae)
Source: PeerJ. 2016 Oct 4;4:e2527. doi: 10.7717/peerj.2527 (PMC5068368; doi:10.7717/peerj.2527)

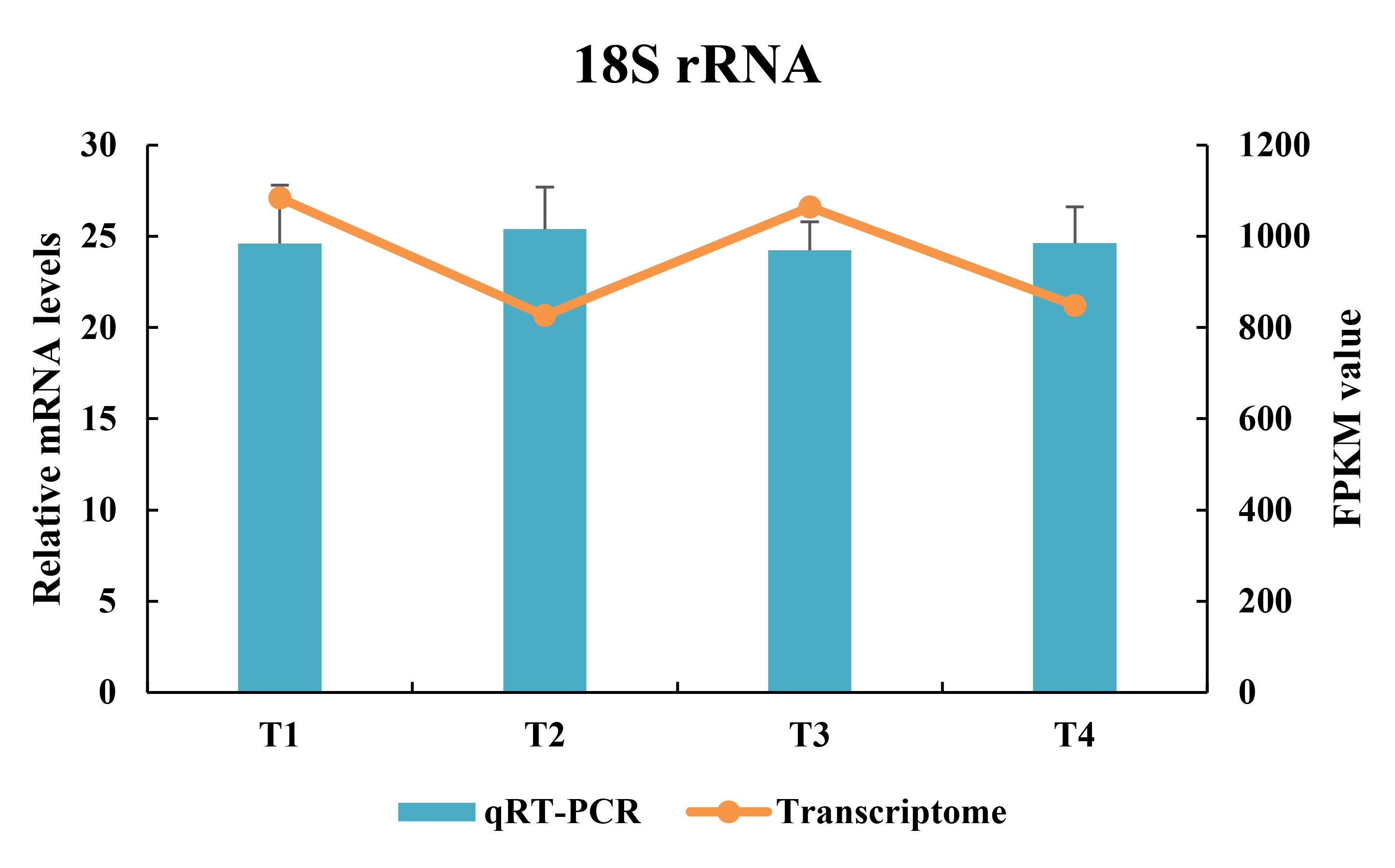

Supplement: Figure S1 [file peerj-04-2527-s001.png]

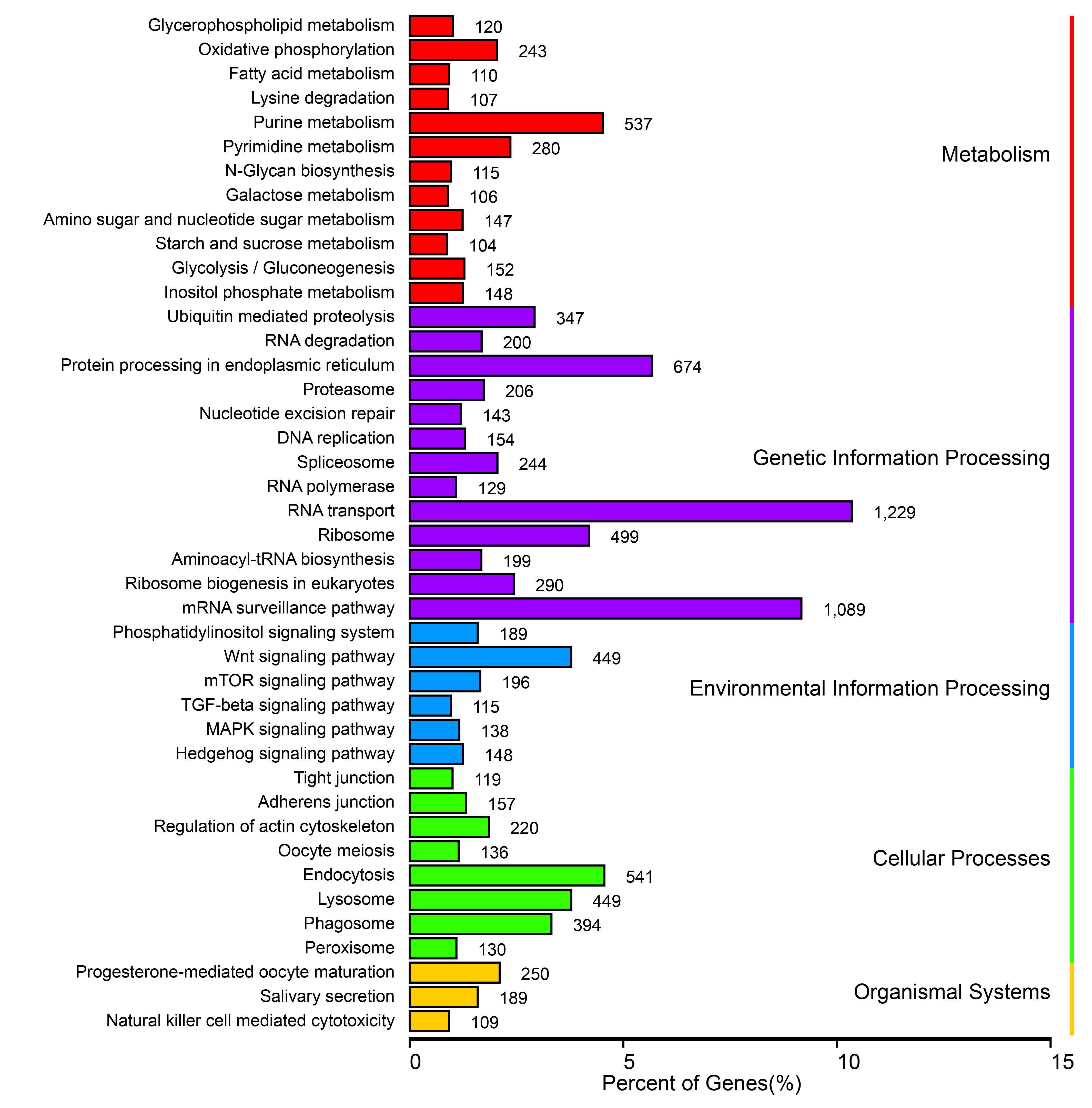

Supplement: Figure S3 [file peerj-04-2527-s003.png]

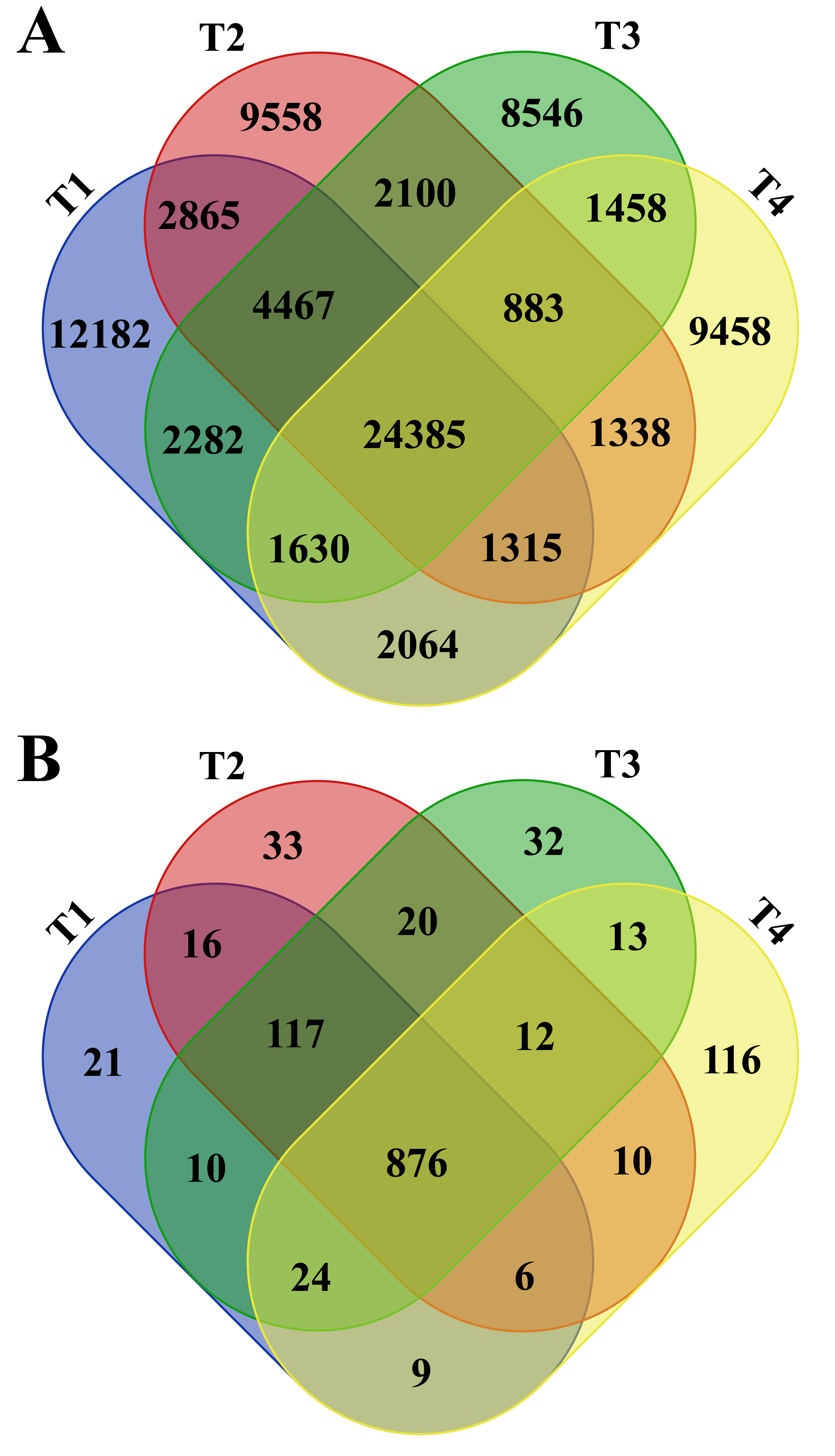

Supplement: Figure S4 — (A) The Venn diagram of the counts of genes with FPKM value more than 1 for four tests T1, T2, T3, and T4 from 84,531 genes. (B) The Venn diagram of the counts of genes with FPKM value more than 60 for four tests T1, T2, T3, and T4 from 1,315 genes. [file peerj-04-2527-s004.png]

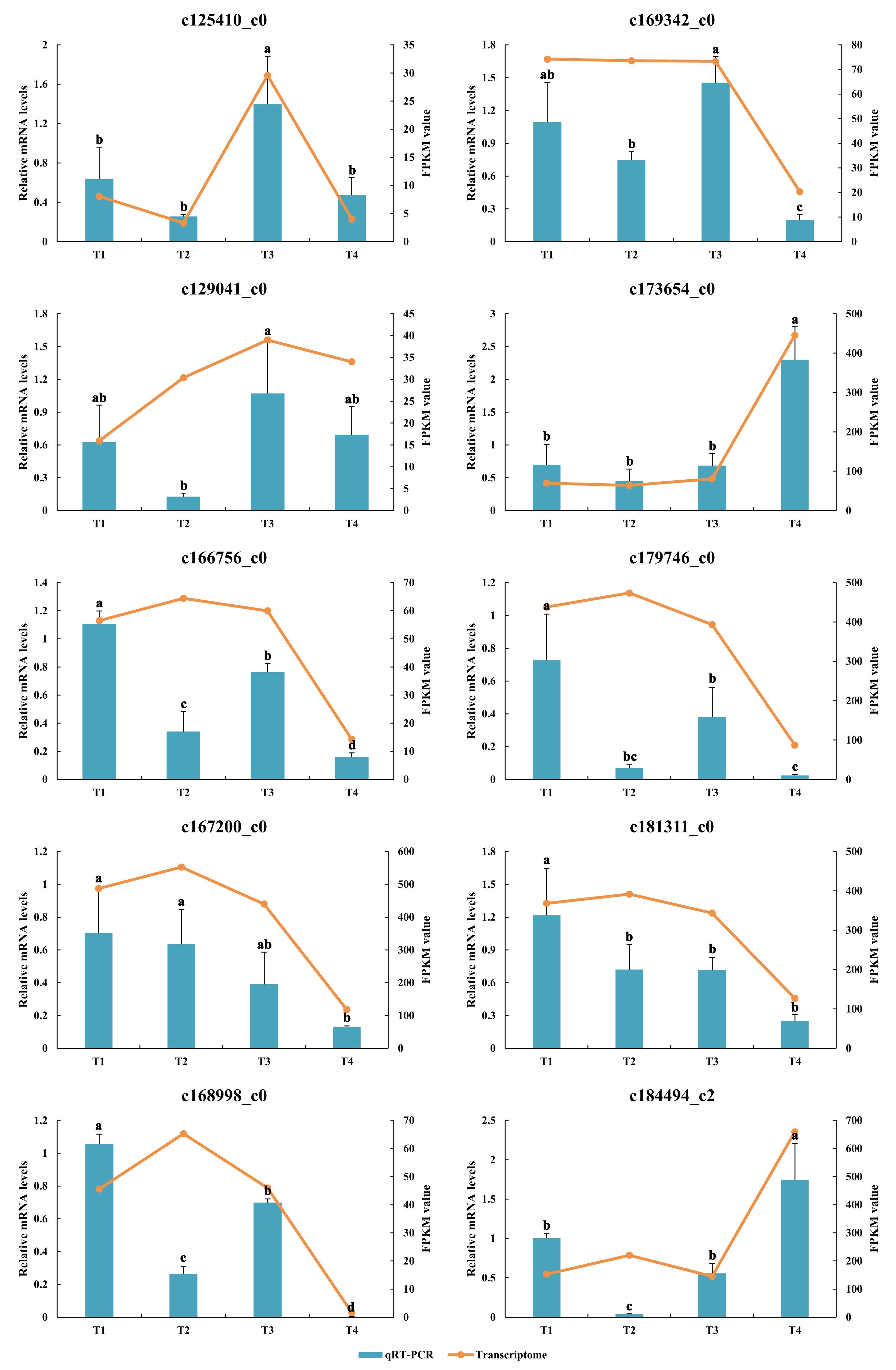

Supplement: Figure S5 — The relative expression of a candidate gene was normalized against 18S ribosomal RNA. [file peerj-04-2527-s005.png]
